# Association of Deepwater Horizon Oil Spill Response and Cleanup Work With Risk of Developing Hypertension

**DOI:** 10.1001/jamanetworkopen.2022.0108

**Published:** 2022-02-23

**Authors:** Richard K. Kwok, W. Braxton Jackson, Matthew D. Curry, Patricia A. Stewart, John A. McGrath, Mark Stenzel, Tran B. Huynh, Caroline P. Groth, Gurumurthy Ramachandran, Sudipto Banerjee, Gregory C. Pratt, Aubrey K. Miller, Xian Zhang, Lawrence S. Engel, Dale P. Sandler

**Affiliations:** 1 Epidemiology Branch, National Institute of Environmental Health Sciences, National Institutes of Health, Department of Health and Human Services, Research Triangle Park, North Carolina; 2 Office of the Director, National Institute of Environmental Health Sciences, National Institutes of Health, Department of Health and Human Services, Bethesda, Maryland; 3 Social & Scientific Systems, Inc, a DLH holdings company, Durham, North Carolina; 4 ICF Inc, Durham, North Carolina; 5 Stewart Exposure Assessments, LLC, Arlington, Virginia; 6 Exposure Assessment Applications, LLC, Arlington, Virginia; 7 Department of Environmental and Occupational Health, Dornsife School of Public Health, Drexel University, Philadelphia, Pennsylvania; 8 Department of Epidemiology and Biostatistics, School of Public Health, West Virginia University, Morgantown; 9 Department of Environmental Health and Engineering, Bloomberg School of Public Health, Johns Hopkins University, Baltimore, Maryland; 10 Department of Biostatistics, UCLA Fielding School of Public Health, University of California, Los Angeles; 11 Division of Environmental Health, School of Public Health, University of Minnesota, Minneapolis; 12 Department of Medicine, University of North Carolina at Chapel Hill, Chapel Hill; 13 Department of Epidemiology, University of North Carolina at Chapel Hill, Chapel Hill

## Abstract

**Importance:**

Exposure to hydrocarbons, fine particulate matter (PM_2.5_), and other chemicals from the April 20, 2010, Deepwater Horizon disaster may be associated with increased blood pressure and newly detected hypertension among oil spill response and cleanup workers.

**Objective:**

To determine whether participation in cleanup activities following the disaster was associated with increased risk of developing hypertension.

**Design, Setting, and Participants:**

This cohort study was conducted via telephone interviews and in-person home exams. Participants were 6846 adults who had worked on the oil spill cleanup (workers) and 1505 others who had completed required safety training but did not do cleanup work (nonworkers). Eligible participants did not have diagnosed hypertension at the time of the oil spill. Statistical analyses were performed from June 2018 to December 2021.

**Exposures:**

Engagement in cleanup activities following the Deepwater Horizon oil spill disaster, job classes, quintiles of cumulative total hydrocarbons exposure level, potential exposure to burning or flaring oil, and estimated PM_2.5_ were examined.

**Main Outcomes and Measures:**

Systolic and diastolic blood pressure measurements were collected during home exams from 2011 to 2013 using automated oscillometric monitors. Newly detected hypertension was defined as antihypertensive medication use or elevated blood pressure since the spill. Log binomial regression was used to calculate prevalence ratios (PR) and 95% CIs for associations between cleanup exposures and hypertension. Multivariable linear regression was used to estimate exposure effects on continuous blood pressure levels.

**Results:**

Of 8351 participants included in this study, 6484 (77.6%) were male, 517 (6.2%) were Hispanic, 2859 (34.2%) were non-Hispanic Black, and 4418 (52.9%) were non-Hispanic White; the mean (SD) age was 41.9 (12.5) years at enrollment. Among workers, the prevalence of newly detected hypertension was elevated in all quintiles (Q) of cumulative total hydrocarbons above the first quintile (PR for Q3, 1.29 [95% CI, 1.13-1.46], PR for Q4, 1.25 [95% CI, 1.10-1.43], and PR for Q5, 1.31 [95% CI, 1.15-1.50]). Both exposure to burning and/or flaring oil and gas (PR, 1.16 [95% CI, 1.02-1.33]) and PM_2.5_ from burning (PR, 1.26 [95% CI, 0.89-1.71]) for the highest exposure category were associated with increased risk of newly detected hypertension, as were several types of oil spill work including cleanup on water (PR, 1.34 [95% CI, 1.08-1.66]) and response work (PR, 1.51 [95% CI, 1.20-1.90]).

**Conclusions and Relevance:**

Oil spill exposures were associated with newly detected hypertension after the Deepwater Horizon disaster. These findings suggest that blood pressure screening should be considered for workers with occupational hydrocarbon exposures.

## Key Points

**Question:**

Is working on oil spill cleanup associated with hypertension risk?

**Findings:**

This cohort study found that participation in the Deepwater Horizon oil spill cleanup was associated with increased risk of subsequent hypertension diagnosis compared with performing support work. Hypertension was also associated with higher cumulative exposure to total hydrocarbons.

**Meaning:**

These findings suggest that oil spill response and cleanup work associated with the Deepwater Horizon disaster may have long-term health consequences.

## Teaser

This cohort study examines whether there was an increased risk of developing hypertension among cleanup workers following the Deep Horizon oil spill disaster.

## Introduction

The Deepwater Horizon (DWH) explosion on April 20, 2010, caused the largest marine oil spill in US history.^1^ Tens of thousands of individuals participated in oil spill response and cleanup (OSRC) work,^2^ which resulted in respiratory exposures to volatile hydrocarbons, such as benzene, toluene, ethylbenzene, xylenes, and n-hexane (BTEX-H), and other hydrocarbons measured as total petroleum hydrocarbons (THC).^3^ Some cleanup workers were also exposed to in situ burning of crude oil and flaring of oil and/or natural gas, which generated inhalable fine particulate matter (PM_2.5_).^4^

Exposure to THC and PM_2.5_ can have deleterious effects on cardiovascular health. Previous studies reported short-term exposure to THC in heavily polluted cities associated with increased risk of cardiovascular death and emergency hospital visits due to hypertension.^5,6^ Occupational exposure to THC from gasoline vapor has been associated with elevated diastolic blood pressure (BP).^7^ Additionally, communities near oil and/or gas production facilities were reported to have higher risks of hypertension compared with other communities.^8^ In rats, THC exposures have been shown to increase BP.^9^ A meta-analyses of short-term exposure to ambient air pollutants demonstrated associations between PM_2.5_ and increased hypertension.^5^

Despite reported cardiovascular effects of THC and PM_2.5_, there is little research on the effects associated with exposures from oil spills.^10^ Most studies of oil spills have examined acute effects, such as respiratory and dermal irritation, headaches, eye irritation, nausea, and dizziness.^2,11^ Few studies have investigated the association between OSRC exposures and adverse cardiovascular outcomes; none have examined BP.^12,13^ Our objective was to address a literature gap by investigating the association of short-term OSRC exposures with BP levels up to 3 years after the DWH disaster.

## Methods

This cohort study was approved by the institutional review board of the National Institute of Environmental Health Sciences. Written informed consent was obtained from all participants during the home exam. This study followed the Strengthening the Reporting of Observational Studies in Epidemiology (STROBE) reporting guideline.

### Study Design and Participants

**Figure 1.**
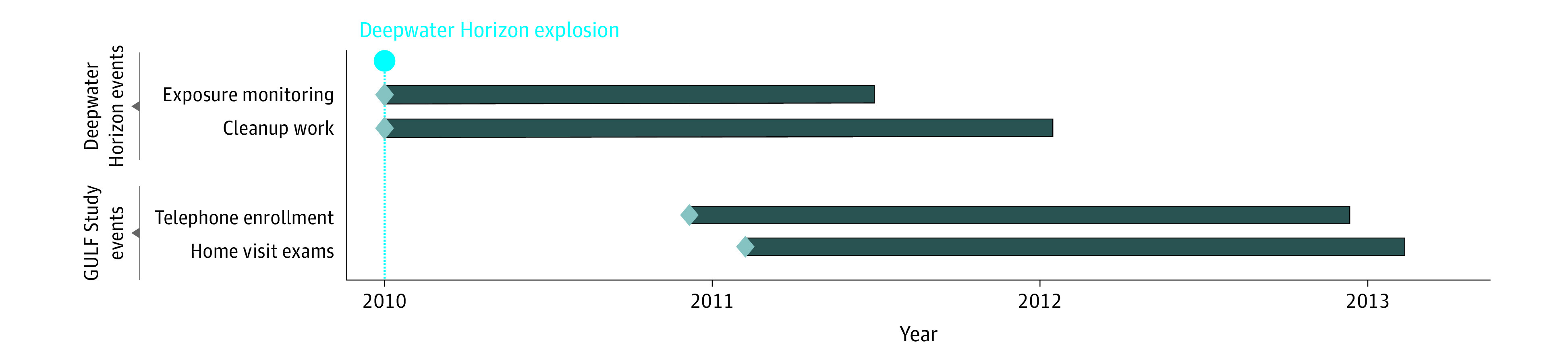
GuLF Study Timeline, 2010-2013

The Gulf Long-Term Follow-Up Study (GuLF Study), is a prospective study of 32 608 OSRC workers following the DWH disaster.^14^ Briefly, persons from across the United States who worked at least 1 day on any activity related to the OSRC (workers) and others who received oil spill safety training but not hired (nonworkers) enrolled from March 2011 to March 2013 (Figure 1). Participants completed a telephone enrollment interview about oil spill jobs and activities, demographics, lifestyle, and health. Following enrollment, a home exam was conducted (May 2011 to May 2013) with participants living in the 5 states bordering the Gulf of Mexico (n = 11 193) to collect BP measures, BMI, and current medications.

Of those completing a home exam, we excluded 2651 (23.7%) who reported being diagnosed with hypertension before the oil spill or were missing information about the timing of the diagnosis. We also excluded 191 (1.7%) with missing BP measurements and/or key adjustment factors. Our final analysis sample included 8351 participants. For analyses of exposures among workers only, we had 6846 participants.

### Oil Spill Response and Cleanup Work Exposure Estimation

Details of the exposure assessment are described elsewhere.^15^ Briefly, exposure assessment was based on work histories obtained from the enrollment interviews^14^ that linked participants to exposure groups (EGs) defined by similarity of their job, activities, or tasks; location of work; and period of work.^15,16^ Based on measurements associated with EGs, participants were assigned THC exposures as an indicator of the crude oil mixture exposure for each activity performed each day worked.^15,16,17,18^ We created 2 measures of estimated cumulative THC exposure: cumulative maximum daily exposure, which summed the maximum daily THC exposure level, and cumulative mean exposure, which summed the mean daily exposure levels. These THC values were categorized into quintiles based on the exposure distribution among workers.

### Other Exposure Metrics

Workers were categorized by industrial hygienists into 6 job classes based on the expected level of THC exposure: response (highest exposure); operations; cleanup on water; decontamination; cleanup on land; and support (lowest exposure).^14,15,16^ Because many workers reported activities in more than one class, workers were assigned the highest relevant job class.

We used self-reported information on activities, location, and timing of work for evaluating exposure to burns. We also modeled air concentrations of PM_2.5_ from flaring at the wellhead, in situ burning (ISB) offshore, and operation of gasoline or diesel-powered engines on the water.^19^ PM_2.5_ from burning activities was estimated from May 15 to July 15, 2010, when ISB and flaring occurred. Emissions data, meteorological data, and source characterizations were inputs in an air dispersion model (AERMOD) to estimate the maximum 1-hour PM_2.5_ air concentrations associated with OSRC activity for specific areas across the Gulf of Mexico.^15,19^ Modeled PM_2.5_ (μg/m^3^) estimates clustered, corresponding to specific locations and activities. Exposure estimates were identified for 2 areas, the hot zone (within 1 nautical mile [nmi] of the wellhead, 1 hour maximum exposure: 545 μg/m^3^); the source (1-5 nmi of the wellhead, 1 hour maximum exposure: 177 μg/m^3^) and 1 activity: ISB (1 hour maximum exposure: 67 μg/m^3^). Engine emissions from vessels or equipment on land were not used because of data uncertainty. We considered all other workers on water (May 15 to July 15, 2010) as low exposed and combined them with the ISB workers because of small numbers (n = 12). We included all other workers (nonwater) as an additional referent category. The maximum PM_2.5_ level experienced by each participant was the exposure metric.^19^

### Outcome Measurements

Systolic and diastolic BP measurements were collected using automated oscillometric monitors during the home exam a few weeks after the enrollment interview (2011-2013). Three BP measurements were taken in a seated position after a rest period with 5 minutes between measurements. BP was calculated as the mean of the last 2 measurements. We defined newly detected hypertension as either participant reported antihypertensive medication use since the spill (40%) or mean measured systolic BP greater than or equal to 140 mm Hg and/or diastolic BP greater than or equal to 90 mm Hg at the time of the home exam (72%), based on contemporaneous hypertension guidelines.^20^ Approximately 12% of our participants reported both antihypertensive medication use and mean measured systolic BP of greater than or equal to 140 mm Hg and/or diastolic BP of greater than or equal to 90 mm Hg. Secondary analyses were performed using hypertension cut points recently issued by the American College of Cardiology (systolic BP greater than or equal to 130 mm Hg and/or diastolic BP greater than or equal to 80 mm Hg).^21^ We also examined BP as a continuous variable.

### Adjustment Factors

Adjustment factors considered were: age (continuous); self-identified gender; self-identified race and ethnicity; body mass index (categorized using NHANES definitions)^22^; educational attainment; and smoking status at enrollment. A directed acyclic graph with these associations is provided (eFigure in the Supplement).

### Statistical Analysis

Characteristics of participants were reported as means and SDs (continuous variables) and percentages (categorical variables), overall, by hypertension status, and among workers. Multivariable log binomial regression was used to estimate prevalence ratios (PRs) and 95% CIs. We evaluated hypertension risk by worker status (worker/non-worker). Among workers, we examined hypertension risk across OSRC job classes with low-exposed workers as the referent group, and separately, risk across quintiles of the 2 cumulative THC exposure metrics, with the lowest quintile as the referent. To evaluate risk associated with burning or flaring exposures, we first compared workers with jobs that exposed them to higher levels of burning oil or flaring natural gas to those without these jobs. Finally, we examined hypertension risk among those with jobs worked in areas with higher PM_2.5_ air concentrations.

Because of the importance of obesity, race and ethnicity, and smoking in hypertension risk, we examined associations between hypertension and cumulative THC exposure in stratified analyses. These models adjusted for age, gender, race and ethnicity, education, smoking status, and obesity but had the stratification variable removed from the model.

Multivariable linear regression was used to examine the association between cumulative THC exposure and continuous systolic and diastolic BP levels. We conducted the analysis 2 ways: (1) including participants on antihypertensive medications and adding 15 mm Hg to their systolic measurements and 10 mm Hg to their diastolic measurements to account for treatment effects,^23^ and (2) excluding participants on antihypertensive medications. We conducted 2 sensitivity analyses to account for correlations between PM_2.5_ and THC exposures. When PM_2.5_ was the primary exposure, we adjusted for cumulative THC exposure. When cumulative THC was the primary exposure, we excluded 665 participants who had PM_2.5_ exposure at the source or hot zone to eliminate the higher concentrations from burning as a source of confounding. Although interpretation of findings was not based on an a priori level of statistical significance, all hypothesis tests were 2-sided and 95% CIs are reported. Tests for exposure-response trends were conducted by modeling the exposures continuously. All statistical analyses were carried out using SAS version 9.4 (SAS Institute) from June 2018 to December 2021.

## Results

### General Characteristics

Characteristics of the 8351 study participants at enrollment are shown in Table 1. Among the 8351 study participants, the mean (SD) age was 41.9 (12.5) years; 6484 (77.6%) were male, 517 (6.2%) were Hispanic, 2859 (34.2%) were non-Hispanic Black, 4418 (52.9%) were non-Hispanic White, 3977 (47.8%) were married, 4747 (56.8%) were former or current smokers, 6017 (72.2%) currently consumed alcohol, 3755 (44.9%) completed high school, 3154 (37.8%) were classified as having a BMI in the obese category, and 3897 (47.6%) had health insurance. Workers and the overall sample were similar across characteristics (Table 1). Compared with those with normal BP, those with hypertension were more likely to be male, non-Hispanic, older, and have higher BMI and lower levels of educational attainment. The 2 groups did not differ in terms of health insurance coverage.

**Table 1.** Participant Characteristics by Hypertension Status, Overall and Among Workers

	Participants, No. (%)				
	All Participants	Overall		Workers only	
		Hypertensive	Not hypertensive	Hypertensive	Not hypertensive
No. of participants	8351	1883	6468	1550	5296
Age, mean (SD), y	41.9 (12.5)	48.3 (12.0)	40.1 (12.1)	47.8 (12.0)	39.5 (11.8)
Gender					
Male	6484 (77.6)	1579 (83.9)	4905 (75.8)	1321 (85.2)	4104 (77.5)
Female	1867 (22.4)	304 (16.1)	1563 (24.2)	229 (14.7)	1192 (22.5)
BMI, mean (SD)	29.0 (6.5)	30.9 (7.0)	28.5 (6.3)	31.1 (7.0)	28.6 (6.2)
Underweight (<18.5)	114 (1.4)	13 (0.7)	101 (1.6)	11 (0.7)	76 (1.4)
Normal (18.5 to <25)	2236 (26.8)	324 (17.2)	1912 (29.6)	256 (16.5)	1525 (28.8)
Overweight (25 to <30)	2847 (34.1)	603 (32.0)	2244 (34.7)	496 (32.0)	1871 (35.3)
Obese (≥30)	3154 (37.8)	943 (50.1)	2211 (34.2)	787 (50.8)	1824 (34.5)
Race and ethnicity					
Hispanic	517 (6.2)	126 (6.7)	391 (6.1)	107 (6.9)	314 (5.9)
Non-Hispanic					
Black	2859 (34.2)	703 (37.3)	2156 (33.3)	573 (37.0)	1792 (33.8)
White	4418 (52.9)	927 (49.2)	3491 (54.0)	767 (49.5)	2856 (53.9)
Other^a^	557 (6.7)	127 (6.7)	430 (6.7)	103 (6.7)	334 (6.3)
Marital status					
Married/living with partner	3977 (47.8)	957 (50.9)	3020 (46.9)	790 (51.1)	2480 (47.0)
Widowed, divorced, or separated	1890 (22.7)	535 (28.5)	1355 (21.0)	434 (28.1)	1096 (20.8)
Never married	2457 (29.5)	387 (20.6)	2070 (32.1)	322 (20.8)	1704 (32.3)
Smoking status					
Never	3604 (43.2)	744 (39.5)	2860 (44.2)	615 (39.7)	2342 (44.2)
Former	1559 (18.7)	416 (22.1)	1143 (17.7)	336 (21.7)	905 (17.1)
Current	3188 (38.2)	723 (38.4)	2465 (38.1)	599 (38.7)	2049 (38.7)
Alcohol consumption					
Never	629 (7.5)	140 (7.4)	489 (7.6)	109 (7.0)	418 (7.9)
Former	1694 (20.3)	456 (24.2)	1238 (19.2)	373 (24.1)	1004 (19.0)
Current	6017 (72.2)	1286 (68.3)	4731 (73.3)	1067 (68.9)	3867 (73.1)
Education					
Less than high school	1734 (20.8)	468 (24.9)	1266 (19.6)	390 (25.2)	1032 (19.5)
High school diploma/GED	2862 (34.3)	663 (35.2)	2199 (34.0)	541 (34.9)	1807 (34.1)
Some college/2-y degree	2518 (30.2)	527 (28.0)	1991 (30.8)	435 (28.1)	1676 (31.7)
4-y college graduate or more	1237 (14.8)	225 (12.0)	1012 (15.7)	184 (11.9)	781 (14.8)
Health care coverage					
Yes, has health insurance	3897 (47.6)	901 (48.7)	2996 (47.3)	730 (47.9)	2381 (45.9)

Abbreviation: BMI, body mass index calculated as weight in kilograms divided by height in meters squared.

^a^ Other races and ethnicities included American Indian or Alaskan Native, Asian, Native Hawaiian or Pacific Islander, or other.

### Hypertension and Oil Spill Exposures

Workers (1550 hypertensive [22.6%] and 5296 nonhypertensive [77.4%]) were not at increased risk of hypertension (PR, 1.04 [95% CI, 0.94-1.14]) compared with nonworkers (333 hypertensive [22.1%] and 1172 nonhypertensive [77.1%]) (Table 2). However, among workers, prevalence ratios for hypertension were elevated for workers assigned to the cleanup work on water (PR, 1.34 [95% CI, 1.08-1.66]), operations (PR, 1.31 [95% CI, 1.06-1.61]), and response (PR, 1.51 [95% CI, 1.20-1.90]) classes compared with support workers with low exposure.

**Table 2.** Hypertension Risk in Relation to Work Exposures^a^

	Participants, No.		PR (95% CI)
	Hypertensive	Nonhypertensive	
**Participant characteristics**			
Full cohort (n = 8351)^b^			
Nonworker	333	1172	1 [Reference]
Worked 1 d on spill	1550	5296	1.04 (0.94-1.14)
Among workers (n = 6846)^b^			
OSRC job classes (in increasing order of exposure)			
Support	81	435	1 [Reference]
Cleanup on land	219	758	1.10 (0.88-1.38)
Decon	202	771	1.24 (0.99-1.56)
Cleanup on water	344	1097	1.34 (1.08-1.66)
Operations	507	1624	1.31 (1.06-1.61)
Response	188	586	1.51 (1.20-1.90)
**Cumulative maximum THC level (ppm-days)^c^**			
Quintile 1	290	1072	1 [Reference]
Quintile 2	285	1078	1.05 (0.93-1.18)
Quintile 3	321	1041	1.29 (1.13-1.46)
Quintile 4	319	1044	1.25 (1.10-1.43)
Quintile 5	326	1036	1.31 (1.15-1.50)
**Cumulative maximum THC level (ppm-days)^d^**			
Quintile 1	281	1081	1 [Reference]
Quintile 2	297	1065	1.19 (1.06-1.32)
Quintile 3	308	1055	1.15 (1.03-1.28)
Quintile 4	332	1031	1.35 (1.19-1.54)
Quintile 5	323	1039	1.29 (1.13-1.47)
Exposure to burning oil and/or gas			
No	1360	4687	1 [Reference]
Yes	161	510	1.16 (1.02-1.33)
PM_2.5_ (μg/m^3^)			
Non-water workers	767	2767	0.89 (0.82-0.98)
Low-exposed water workers^e^	519	1646	1 [Reference]
Source (1 hr maximum: 177 μg/m^3^)	131	46	1.07 (0.92-1.25)
Hot zone (1 hr maximum: 545 μg/m^3^)	23	65	1.26 (0.89-1.77)

Abbreviations: OSRC, oil spill response and cleanup; PM_2.5_, fine particulate matter; PR, prevalence ratios; THC, total petroleum hydrocarbons.

^a^ Multivariable log binomial regression models adjusted for age, gender, race/ethnicity, education, smoking status, and obesity.

^b^ 34 workers had no exposure information due to starting work after June 30, 2011, or not enough information to assign exposure.

^c^ Cumulative daily maximum total hydrocarbon exposure levels; quintile 1 (0.02-14.66 ppm-days); quintile 2 (8.88-24.99 ppm-days); quintile 3 (25.00-51.30 ppm-days); quintile 4 (51.36-92.80 ppm-days); quintile 5 (92.86-687.42 ppm-days).

^d^ Cumulative daily mean total hydrocarbon exposure levels; quintile 1 (0.02-8.87 ppm-days); quintile 2 (14.72-43.44 ppm-days); quintile 3 (43.48-92.34 ppm-days); quintile 4 (92.39-198.18 ppm-days); quintile 5 (198.34-1053.12 ppm-days).

^e^ Includes other water workers and in situ burn workers (PM_2.5_ level: 1 hour maximum: 67 μg/m^3^).

Among workers, cumulative maximum THC exposure in the highest 3 quintiles (Q3: 25.00-51.30 ppm-days; Q4: 51.36-92.80 ppm-days; Q5: 92.86-687.42 ppm-days) was associated with increased hypertension risk (PR for Q3, 1.29 [95% CI, 1.13-1.46]; PR for Q4, 1.25 (95% CI, 1.10-1.43); and PR for Q5, 1.31 [95% CI, 1.15-1.50]) (Table 2). Associations were similar for estimated cumulative mean THC (Table 2) and after excluding 665 participants who had the higher PM_2.5_ exposure from working in the source and/or hot zone (eTable 1 in the Supplement).

OSRC workers exposed to burning oil and/or flaring natural gas had an increased hypertension risk (PR, 1.16 [95% CI, 1.02-1.33]) (Table 2). For workers in the source and/or hot zone areas with higher PM_2.5_ air concentrations, the magnitude of risk was similar but confidence limits were imprecise (PR, 1.26 [95% CI, 0.89-1.77]). In a sensitivity analysis adjusting for cumulative THC exposure, results for PM_2.5_ were similar (eTable 1 in the Supplement).

**Figure 2.**
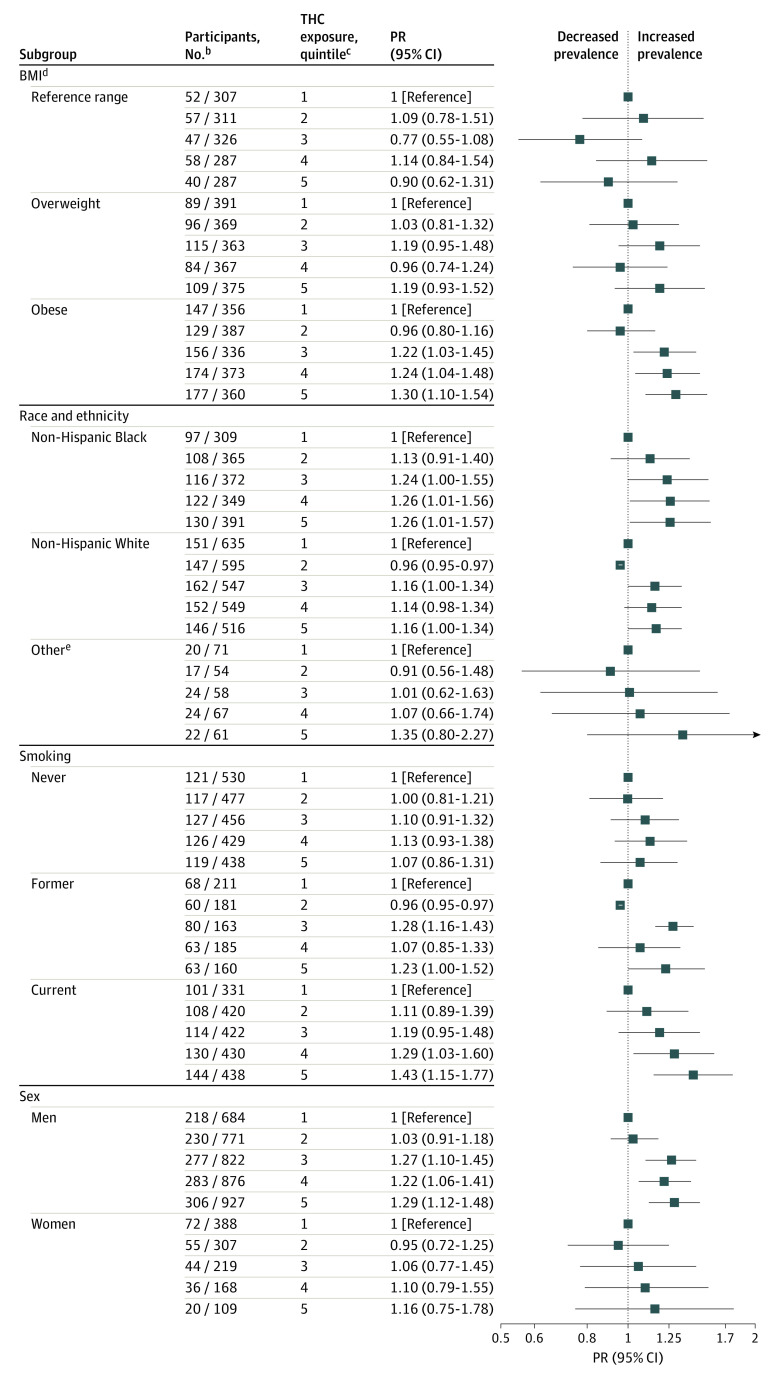
Hypertension Risk Associated With THC Exposure Levels, Stratified by BMI, Gender, Smoking Status, and Race/Ethnicity, Among Workers^a^ ^a^Multivariable log binomial regression models adjusted for age, gender, race/ethnicity, education, smoking status, and obesity (except when it is the stratification variable). ^b^Participants with hypertension/participants without hypertension. ^c^Cumulative daily maximum total hydrocarbon exposure levels; quintile 1 (0.02-14.66 ppm-days); quintile 2 (8.88-24.99 ppm-days); quintile 3 (25.00-51.30 ppm-days); quintile 4 (51.36-92.80 ppm-days); quintile 5 (92.86-687.42 ppm-days). ^d^Body mass index calculated as weight in kilograms divided by height in meters squared; underweight not included in the model. ^e^Other races and ethnicities included American Indian or Alaskan Native, Asian, Native Hawaiian or Pacific Islander, or other.

The pattern of association between THC and hypertension was most clear among participants with obesity (Figure 2), with elevated PRs in the top 3 quintiles of cumulative maximum THC compared with the lowest quintile (PR_Q3_, 1.22 [95% CI, 1.03-1.45]; PR for Q4, 1.24 [95% CI, 1.04-1.48]; PR for Q5, 1.30 [95% CI, 1.10-1.54]. In analyses stratified by race and ethnicity, non-Hispanic Black participants had elevated hypertension in all quintiles compared with the lowest quintile of cumulative maximum THC exposure (PR for Q2, 1.13 [95% CI, 0.91-1.40]; PR for Q3, 1.24 [95% CI, 1.00-1.55]; PR for Q4, 1.26 [95% CI, 1.01-1.56]; and PR for Q5, 1.26 [95% CI, 1.01-1.57]). For non-Hispanic White participants, the hypertension prevalence ratios were elevated only in the top 3 quintiles (PR for Q3, 1.16 [95% CI, 1.00-1.34]; PR for Q4, 1.14 [95% CI, 0.98-1.34]; and PR for Q5, 1.16 [95% CI, 1.00-1.34]). Among current smokers, hypertension prevalence ratios increased with increasing levels of cumulative maximum THC (PR for Q2, 1.11 [95% CI, 0.89-1.39]; PR for Q3, 1.19 [95% CI, 0.95-1.48]; PR for Q4, 1.29 [95% CI, 1.03-1.60]; and PR for Q5, 1.43 [95% CI, 1.15-1.77]). Trends were less clear for former smokers: hypertension risk in the higher quintiles of cumulative maximum THC exposure was increased, but not linearly (PR for Q3, 1.28 [95% CI, 1.16-1.43]; PR for Q4, 1.07 [95% CI, 0.85-1.33]; and PR for Q5, 1.23 [95% CI, 1.00-1.52]). Although patterns of association between THC and hypertension were similar for men and women, associations were more pronounced among men with elevated prevalence ratios in the top 3 quintiles of exposure compared with the lowest (PR_Q3_, 1.27 [95% CI, 1.10-1.45]; PR_Q4_, 1.22 [95% CI, 1.06-1.41]; and PR_Q5_, 1.29 [95% CI, 1.12-1.48]).

### Blood Pressure and THC Exposure

In multivariable linear regression analysis of continuous BP measurements, both systolic and diastolic BP increased with increasing levels of cumulative maximum THC exposure, although the trend was clearer for diastolic BP (Table 3). Associations with diastolic BP were attenuated after excluding participants on BP medications. Some estimates from analyses using the more recent hypertension definition (systolic ≥130 mm Hg, diastolic ≥ 80 mm Hg) PR were attenuated but the patterns of association did not change appreciably (eTable 2 in the Supplement).

**Table 3.** Association of Blood Pressure Levels and Cumulative Maximum THC Exposure^a^

Blood pressure	Cumulative maximum THC^b^	Change in blood pressure (n = 6812)	Change in blood pressure with no medication (n = 6195)
		Estimate (95% CI)^c^	Estimate (95% CI)^c^
Systolic	Quintile 1	[Reference]	[Reference]
	Quintile 2	−0.26 (−1.37 to 0.85)	−0.49 (−1.54 to 0.57)
	Quintile 3	0.56 (−0.57 to 1.68)	−0.18 (−1.24 to 0.89)
	Quintile 4	1.36 (0.23 to 2.49)	0.85 (−0.22 to 1.92)
	Quintile 5	1.02 (−0.13 to 2.15)	0.32 (−0.76 to 1.40)
	Trend	NA	NA
Diastolic	Quintile 1	[Reference]	[Reference]
	Quintile 2	0.14 (−0.67 to 0.94)	−0.007 (−0.79 to 0.78)
	Quintile 3	0.86 (0.06 to 1.67)	0.30 (−0.49 to 1.10)
	Quintile 4	1.26 (0.45 to 2.08)	0.86 (0.06 to 1.65)
	Quintile 5	1.47 (0.64 to 2.29)	1.12 (0.32 to 1.93)
	Trend	NA	NA

Abbreviations: THC, total petroleum hydrocarbons; NA, not applicable.

^a^ Multivariable linear regression, controlling for gender, age, BMI, race, education, and smoking status.

^b^ Quintile 1 (0.02-14.66 ppm-days); quintile 2 (14.72-43.44 ppm-days); quintile 3 (43.48-92.34 ppm-days); quintile 4 (92.39-198.18 ppm-days); quintile 5 (198.34-1053.12 ppm-days).

^c^ β coefficient associated with a 1 mm Hg increase in systolic or diastolic pressure.

## Discussion

To our knowledge, this is the first study to investigate the association of OSRC exposures from the DWH disaster and hypertension. Among workers, higher exposures to oil spill–related chemicals were associated with elevated risk of newly detected hypertension, especially among workers with obesity and those who were non-Hispanic Black, current smokers, and men—risk factors traditionally associated with hypertension.^5^ Those with OSRC jobs with higher THC exposures were also more likely to have newly detected hypertension after the spill. Exposure to burning of oil and/or flaring of oil or natural gas was associated with newly detected hypertension risk in analyses that considered exposure based on proximity to burn sites or modeled PM_2.5_ estimates. A lack of clear differences in hypertension risk between workers and nonworkers may be associated with previously noted concerns that those who did not participate in OSRC efforts may have been less healthy than those able to work.^14^

Components of THC, including BTEX-H, have been associated with a range of negative health outcomes related to hypertension. Studies of oil refinery workers and residents living in communities surrounding oil and/or gas refineries have reported increased risk of hypertension.^8^ Previous studies have shown that oil exposure from prolonged cleanup activity induced oxidative stress in OSRC workers up to at least 1 year following the last exposure,^24^ which could lead to the development of elevated BP and hypertension.^25^ Other occupational groups such as taxi drivers exposed to BTEX were reported to have higher prevalence of hypertension.^26^ DWH OSRC workers were also exposed to burning oil and combustion byproducts such as PM_2.5_. Several experimental studies and epidemiologic studies in the general population have demonstrated how PM_2.5_ increased risk factors that contribute to the development of atherosclerosis, which can lead to hypertension.^27,28^ More directly, studies have reported significant associations between exposure to ambient PM_2.5_ and increased odds of hypertension in the general population.^5^

In addition to having OSRC-related exposures, the GuLF Study population is at risk for hypertension because of lifestyle and socioeconomic factors. For example, 37.8% were classified as having a BMI in the obese category, which is slightly higher than the NHANES age-adjusted prevalence of obesity (36.3%).^22^ Additionally, a smaller proportion of our study population obtained a high school diploma (44.9%) compared with the NHANES 2011-2014 survey population (60.0%).^22^ Much of the cohort and the general population from which OSRC workers were drawn is medically underserved. Only 47.6% of the participants reported having some form of health insurance coverage compared with 82% in the NHANES survey.^29^ This lack of medical care access or inability to afford some prescribed medications is consistent with the observation that only 38% of those who developed hypertension after the oil spill were classified as such because they were taking antihypertensive medications at the time of the home exam. An additional 12% reported taking or being prescribed antihypertensive medications but still had BP measurements that exceeded the treatment guidelines in use at the time of the home exam. Adjusting for education, smoking, and obesity, however, did not alter associations between OSRC exposures and hypertension.

We considered the possibility that experiencing other symptoms during cleanup may have led some workers to be differentially diagnosed with hypertension. However, the frequency of health insurance coverage among workers and the full cohort did not differ, and insurance coverage was not associated with how hypertension was detected (antihypertensive medication use, measured elevated BP, or both). Furthermore, the proportion of participants categorized as hypertensive by medication only, by elevated BP only or by both approaches was identical for the full sample and among workers only, suggesting that workers were not differentially diagnosed.

### Limitations and Strengths

This study had a few limitations. There was a potential for selection bias when we reduced the study sample from 32 608 participants in the GuLF study nationwide, to the 11 193 participants from the 5 Gulf states who participated in the home exam, to the 8351 participants who did not have hypertension prior to the spill or were missing data on adjustment factors or prior hypertension, to the final sample of 6846 workers with exposure data. However, the characteristics of those who participated in the home exam are similar to those of the larger cohort suggesting that selection bias is unlikely (eTable 3 in the Supplement).

Although we excluded participants with hypertension diagnosed before the oil spill, we cannot be sure that hypertension identified at the home exam was incident rather than simply undiagnosed before the spill. Nevertheless, undiagnosed hypertension was equally likely among workers and nonworkers, resulting in nondifferential misclassification. Our results should be confirmed in longitudinal analyses with repeated measures to determine whether the associations persist.

Because THC exposure was based in part on self-reported work activities performed during the OSRC, misclassification of exposures is possible especially given the large geographic area covered. The quantitative estimates were associated with some degree of uncertainty.^3^ Additionally, there is the possibility of recall bias because some participants had to recall work activities from nearly 3 years prior. However, this association would be nondifferential because participants across all exposure categories enrolled across the duration of study enrollment. There is the possibility of a healthy worker effect where jobs at the source were more likely assigned to workers in better overall health. Finally, the exposures examined did not identify the specific etiologically relevant chemical agent(s).

This study also had some strengths. It is one of the largest studies examining the associations of THC exposure with newly detected hypertension following an oil spill. It used a comprehensive set of THC measurements taken during the DWH OSRC. The large sample size of this study allowed stratified analyses of subgroups of interest. Furthermore, automated oscillometric BP monitors provide more accuracy and are less prone to measurement error and digit preference than manual sphygmomanometry.^30^

## Conclusions

This cohort study found that workers with higher cumulative THC exposure during the Deepwater Horizon OSRC effort were more likely to have newly detected hypertension after the spill. Exposure to burning oil or flaring of oil and/or natural gas during the OSRC was also associated with increased hypertension risk. Future studies should examine whether these associations persist over time.

## Notes

Supplement. **eTable 1.** Hypertension Risk in Relation to Work Exposures **eTable 2.** Hypertension Prevalence in Relation to Exposure using New Hypertensive Guidelines of 130/80 mmHg **eTable 3.** Participant Characteristics Among Full Cohort, Home Exam, Analytic Sample, and Analytic Sample of Workers **eFigure.** Directed Acyclic Graph Showing Potential Confounders for the Relationship Between Oil Spill Response and Cleanup Exposures and Hypertension
