# Error in Table 2 and Figure 1

**DOI:** 10.1001/jamanetworkopen.2022.6792

**Published:** 2022-03-18

In the Original Investigation titled “Association of Deepwater Horizon Oil Spill Response and Cleanup Work With Risk of Developing Hypertension,”^1^ published February 23, 2022, there was an error in listing cumulative maximum THC level twice in Table 2. The second listing should have instead been “Cumulative mean THC level.” Additionally, in Figure 1, the timeline was adjusted to indicate that telephone enrollment started in March 2011 and home visits started in May 2011. This article has been corrected.^1^
