# Error in Table 2 and Supplement

**DOI:** 10.1001/jamanetworkopen.2022.43198

**Published:** 2022-11-03

In the Original Investigation titled “Association of Deepwater Horizon Oil Spill Response and Cleanup Work With Risk of Developing Hypertension,”^1^ published February 23, 2022, the same error appeared in both Table 2 and eTable 1 in the Supplement. In both tables, the row “Source (1 hr maximum: 177 μg/m^3^)” incorrectly listed the value for the nonhypertensive group as 46 instead of 446. This article has been corrected.^1^
