# Supplementary material for: Association of Deepwater Horizon Oil Spill Response and Cleanup Work With Risk of Developing Hypertension
Source: JAMA Netw Open. 2022 Feb 23;5(2):e220108. doi: 10.1001/jamanetworkopen.2022.0108 (PMC8867245; doi:10.1001/jamanetworkopen.2022.0108)
Supplement: Supplement. — eTable 1. Hypertension Risk in Relation to Work Exposures eTable 2. Hypertension Prevalence in Relation to Exposure using New Hypertensive Guidelines of 130/80 mmHg eTable 3. Participant Characteristics Among Full Cohort, Home Exam, Analytic Sample, and Analytic Sample of Workers eFigure. Directed Acyclic Graph Showing Potential Confounders for the Relationship Between Oil Spill Response and Cleanup Exposures and Hypertension [file jamanetwopen-e220108-s001.pdf]

Supplemental Online Content

Kwok RK, Jackson WB II, Curry MD, et al. Association of Deepwater Horizon oil spill response and cleanup work with risk of developing hypertension. *JAMA Netw Open.* 2022;5(2):e220108. doi:10.1001/jamanetworkopen.2022.0108

eTable 1. Hypertension Risk in Relation to Work Exposures

eTable 2. Hypertension Prevalence in Relation to Exposure Using New Hypertensive Guidelines of 130/80 mmHg

eTable 3. Participant Characteristics Among Full Cohort, Home Exam, Analytic Sample, and Analytic Sample of Workers

eFigure. Directed Acyclic Graph Showing Potential Confounders for the Relationship Between Oil Spill Response and Cleanup Exposures and Hypertension

This supplemental material has been provided by the authors to give readers additional information about their work.

© 2022 Kwok RK et al. *JAMA Network Open.*

eTable 1. **Hypertension Risk in Relation to Work Exposures [a]**

	Hypertensive (n)	Non-Hypertensive (n)	PR (95% CI)
**Among Workers[b]**	**N=6,846**		
**Cumulative$_{max}$THC level[c] (ppm-days)[d]**			
Quintile 1	287	1,068	ref
Quintile 2	280	1,058	1.05 (0.93-1.18)
Quintile 3	304	986	1.27 (1.12-1.45)
Quintile 4	274	903	1.26 (1.10-1.44)
Quintile 5	239	732	1.35 (1.18-1.55)
**$PM_{2.5}$ (µg/m^3)[e]**			
Non-water workers	767	2,767	0.97 (0.88-1.07)
Low exposed water workers[f]	519	1,646	ref
Source (1 hr$_{max}$: 177 µg/m^3)	131	46	1.05 (0.89-1.23)
Hotzone (1 hr$_{max}$: 545 µg/m^3)	23	65	1.22 (0.87-1.72)

[a] Multivariable log binomial regression models adjusted for age, gender, race/ethnicity, education, smoking status, and obesity.

[b] 34 workers had no exposure information due to starting work after June 30,2011 or not enough information to assign exposure.

[c] Cumulative daily maximum total hydrocarbon exposure levels; Quintile 1 (0.02-14.66 ppm-days); Quintile 2 (8.88-24.99 ppm-days); Quintile 3 (25.00-51.30 ppm-days); Quintile 4 (51.36-92.80 ppm-days); Quintile 5 (92.86-687.42 ppm-days)

[d] Excluded the 665 participants who had $PM_{2.5}$ exposure at the source/hotzone

[e] Additionally adjusted for cumulative daily maximum total hydrocarbon exposure.

[f] Includes other water workers and "*in situ* burn" workers ($PM_{2.5}$ level: 1 hr$_{max}$: 67 µg/m^3)

© 2022 Kwok RK et al. *JAMA Network Open.*

eTable 2. Hypertension Prevalence in Relation to Exposure using New Hypertensive Guidelines of 130/80 mmHg[a]

	Hypertensive (n)	Non-Hypertensive (n)	PR (95% CI)
**Full Cohort[b]**	**n=8,351**		
Nonworker	635	870	ref
Worked 1 day on Spill	3,027	3,819	1.02 (0.96-1.08)
**Among Workers[b]**	**N=6,846**		
**OSRC Exposure Groups**			
Support	182	334	ref
Clean-up on Land	298	579	0.96 (0.84-1.09)
Decon	405	568	1.09 (0.95-1.24)
Clean-up on Water	678	763	1.16 (1.03-1.30)
Operations	972	1,159	1.10 (0.98-1.24)
Response	371	403	1.22 (1.07-1.39)
**Cumulative$_{max}$THC level[c,d] (ppm-days)**			
Quintile 1	561	801	ref
Quintile 2	565	798	1.05 (0.97-1.12)
Quintile 3	586	776	1.11 (1.03-1.20)
Quintile 4	639	724	1.17 (1.10-1.24)
Quintile 5	655	707	1.20 (1.12-1.30)
**Cumulative$_{ave}$THC level[e, f] (ppm-days)**			
Quintile 1	552	810	ref
Quintile 2	576	786	1.11 (1.04-1.18)
Quintile 3	583	780	1.09 (1.02-1.16)
Quintile 4	645	718	1.20 (1.13-1.27)
Quintile 5	650	712	1.19 (1.10-1.28)
**Exposure to burning oil/gas**			
No	2,653	3,394	ref
Yes	321	350	1.11 (1.02-1.19)
**$PM_{2.5}$ (µg/m^3)**			

© 2022 Kwok RK et al. *JAMA Network Open.*

Non-water workers	1,496	2,038	0.94 (0.89-0.99)
Low exposed water workers[g]	994	1,171	ref
Source (1 hr_{max}: 177 µg/m^3)	267	310	1.06 (0.96-1.16)
Hotzone (1 hr_{max}: 545 µg/m^3)	47	41	1.26 (1.05-1.52)

[a] Multivariable log binomial regression models adjusted for age, gender, race/ethnicity, education, smoking status, and obesity.

[b] 34 workers had no exposure information due to starting work after June 30th 2011 or not enough information to assign exposure.

[c] Cumulative daily average total hydrocarbon exposure levels

[d] Quintile 1 (0.02-14.66 ppm-days); Quintile 2 (8.88-24.99 ppm-days); Quintile 3 (25.00-51.30 ppm-days); Quintile 4 (51.36-92.80 ppm-days); Quintile 5 (92.86-687.42 ppm-days)

[e] Cumulative daily maximum total hydrocarbon exposure levels

[f] Quintile 1 (0.02-8.87 ppm-days); Quintile 2 (14.72-43.44 ppm-days); Quintile 3 (43.48-92.34 ppm-days); Quintile 4 (92.39-198.18 ppm-days); Quintile 5 (198.34-1053.12 ppm-days)

[g] Includes unexposed water workers and "*in situ* burn" workers (PM 2.5 level: 1 hr_{max}: 67 µg/m^3)

© 2022 Kwok RK et al. *JAMA Network Open.*

eTable 3. Participant Characteristics among Full Cohort, Home Exam, Analytic Sample, and Analytic Sample of Workers.

	**Full Cohort**	**Home Exam**	**Analysis Sample**	**Analysis Sample of Workers with Exposure Information**
**Number of participants (No.)**	32,608	11,193	8,351	6,846
**Age (years)** (mean ± SD)	42.8±12.8	43.9±13.0	41.9±12.5	41.4±12.4
**Gender** (%)				
Male	81	78	78	79
Female	19	22	22	21
**BMI (kg/m^2)** (mean ± SD)	28.4±6.0	28.8±6.0	29.0±6.5	29.1±6.5
Underweight (<18.5) (%)	1	1	1	1
Normal (18.5-<25)	27	25	27	26
Overweight 25-<30)	41	39	34	35
Obese (30+)	31	35	38	38
**Race/Ethnicity** (%)				
Non-Hispanic white	62	53	53	53
Non-Hispanic black	22	34	34	35
Hispanic	7	6	6	6
Other/multi-racial	9	7	7	6
**Marital Status** (%)				
Married/Living with partner	57	50	48	48
Widowed/Divorced/Separated	19	23	23	22
Never married	24	27	29	30
**Smoking Status** (%)				
Never	48	42	43	43
Former	22	21	19	18

© 2022 Kwok RK et al. *JAMA Network Open.*

Current	30	37	38	39
**Alcohol Consumption** (%)				
Never	6	7	8	8
Former	18	21	20	20
Current	76	71	72	72
**Education** (%)				
Less than high school	16	21	21	21
High school diploma/GED	30	34	34	34
Some college/2-year degree	30	30	30	31
4-year college graduate or more	24	15	15	14
**Health Care Coverage** (%)[a]				
Yes, has health insurance		50	48	46

[a] Health care coverage question only asked during home exam.

© 2022 Kwok RK et al. *JAMA Network Open.*

eFigure. Directed Acyclic Graph Showing Potential Confounders for the Relationship Between Oil Spill Response and Cleanup Exposures and Hypertension

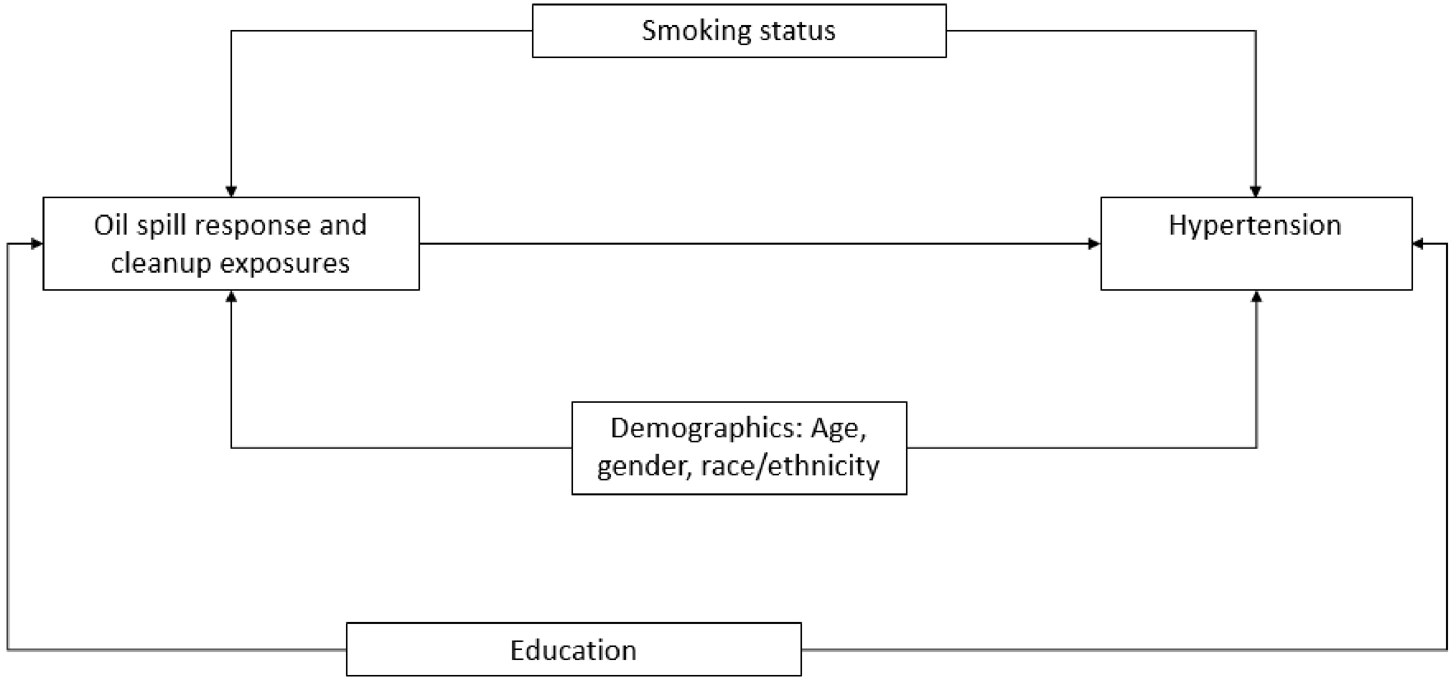

Supplemental Figure 1. Directed acyclic graph showing potential confounders for the relationship between oil spill response and cleanup exposures and hypertension.

© 2022 Kwok RK et al. *JAMA Network Open.*
